# A Novel Ferroptosis-Related Gene Signature for Prognosis Prediction in Ewing Sarcoma

**DOI:** 10.1155/2022/6711629

**Published:** 2022-08-22

**Authors:** Runhan Zhao, Zefang Li, Yanran Huang, Chuang Xiong, Chao Zhang, Hao Liang, Jingtao Xu, Xiaoji Luo

**Affiliations:** ^1^Department of Orthopedics, The First Affiliated Hospital of Chongqing Medical University, Yuzhong, Chongqing, China; ^2^Orthopedic Laboratory of Chongqing Medical University, Yuzhong, Chongqing, China; ^3^Department of Orthopedics, Qianjiang Central Hospital of Chongqing, Qianjiang, Chongqing, China

## Abstract

Ferroptosis, as a form of programmed cell death independent of apoptosis, has been demonstrated that plays a major role in tumorigenesis and cancer treatment. A comprehensive analysis of ferroptosis-related genes (FRGs) may lead to a novel choice for the treatment of Ewing sarcoma (ES). Here, 148 differentially expressed FRGs (DEFRGs) were identified between normal and ES tissue. And the GO and KEGG analyses of DEFRGs indicated that these genes were enriched in cancer and immune-related signaling pathways. Then, the GSE17679 cohort was randomly divided into train and test cohorts. Based on the train cohort, AURKA, RGS4, and RIPK1 were identified as key genes through the univariate Cox regression analysis, the random survival forest algorithm, and the multivariate Cox regression analysis and utilized to establish a prognostic FRG signature. The validation results demonstrated that the gene signature has not only excellent prediction performance and generalization ability but is also good at predicting the response of immunotherapy and chemotherapy. Subsequent analysis indicated that all 3 key genes play key roles in tumor immunity and prognosis of ES. Of these, AURKA was highly associated with EWSR1, which was verified by a single-cell dataset (GSE130019). Therefore, the 3 genes may be potential therapeutic targets for ES. At the end of this study, we also constructed an accurate nomogram that helps clinicians to assess the survival time of ES patients. In conclusion, our study constructed an excellent gene signature, which is helpful in improving the prognosis of ES patients.

## 1. Introduction

Ewing sarcoma (ES) is the second most common bone or soft-tissue tumor affecting children, adolescents, and young adults; is characterized by pathognomonic FET/ETS gene fusions (85% of cases are EWSR1/FLI1); and is an invasive tumor with characters of early metastatic spread, high recurrence, and low 5-year survival [1–3]. Although the treatment of localized disease has been demonstrated effectively, the long-term survival of patients with metastatic or relapsed ES remains unacceptably low [4, 5]. A British cohort of patient study reported that the 5-year survival rate of patients with ES was only 55% [6]. Coupled with lacking reliable statistical tools, the diagnosis and treatment of ES remain a challenge [7].

Ferroptosis, as a form of programmed cell death independent of apoptosis, is a process of cytological changes caused by the accumulation of iron-dependent lipid hydroperoxide [8]. Studies have demonstrated that ferroptosis plays a role in various tumors, such as prostate cancer, renal cell carcinoma, head and neck cancer, and soft tissue sarcoma [9–12]. Additionally, there are also many studies that revealed that ferroptosis plays a role in tumor suppression by regulating metabolic processes and promoting cell death [13, 14]. With an increased understanding of ferroptosis in recent years, the induction of ferroptosis has gradually emerged as a promising therapeutic option, especially for malignant tumors that are resistant to traditional treatments [15, 16]. A deeper understanding of the mechanism of ferroptosis in ES may develop novel treatments for ES. Hence, we conducted this study, which aimed to find key genes from ferroptosis-related genes (FRGs) and construct an accurate prognostic FRG signature.

In this study, we first randomly divided the GSE17679 dataset into train and test cohorts. Then, based on the train cohort, 3 key FRGs were screened out by utilizing three analytical methods (univariate Cox regression analysis, random survival forest algorithm, and multivariate Cox regression analysis). Next, we used the 3 genes to establish a prognostic FRG signature, which has excellent prediction performance and generalization ability. Subsequent analysis demonstrated that the gene signature also owns potential applications in predicting the response of immunotherapy and chemotherapy, and the 3 pivotal genes were potential therapeutic targets. Therefore, our study is helpful in improving the prognosis of ES.

## 2. Materials and Methods

### 2.1. Data Collection and Preprocessing

We downloaded the GSE17679 [17] dataset, including gene expression profiles and clinical data of 88 ES samples and gene expression profiles of 18 normal tissue, from the Gene Expression Omnibus (GEO, https://www.ncbi.nlm.nih.gov/geo/) database. Meanwhile, we also downloaded the GSE63157 dataset [18] (including gene expression profiles and clinical data of 85 ES samples) from the GEO database and the TCGA-SARC dataset from the UCSC browser (including gene expression profiles and clinical data of 255 soft tissue sarcoma samples, https://xena.ucsc.edu/) as external validation cohorts. Additionally, in order to explore the correlation between EWSR1/FLI1 and key genes, we downloaded a single-cell dataset, GSE130019 [19], and processed it by the “Seurat” R package. Doxycycline can inhibit the expression of EWSR1/FLI1, and the GSE130019 dataset contains the gene expression profile of A673 cells during the period of continuous doxycycline use for 7 days and drug withdrawal for 15 days.

### 2.2. Cell Lines and Cell Culture

The human ES cell line (A673) and the human bone marrow stroma cell line (HS5) were obtained from the American Type Culture Collection (ATCC, USA). The cells were grown in DMEM (HyClone; Cytiva) supplemented with 10% FBS (Shanghai ExCell Biology, Inc.) and 1% penicillin-streptomycin (100 IU/ml; HyClone; Cytiva) at 37°C in 5% CO_2_.

### 2.3. Identification of Differentially Expressed FRGs and Functional Annotation

We collected the list of FRGs from a published article [20] and the FerrDb database (http://www.zhounan.org/ferrdb) [21]. Then, we used the “limma” R package to perform the differential gene expression analysis between normal and tumor tissue, and FRGs with a false discovery rate (FDR) < 0.05 were considered as differentially expressed FRGs (DEFRGs). Then, to explore the biological function of DEFRGs, we performed Gene Ontology (GO) and Kyoto Encyclopedia of Genes and Genomes (KEGG) enrichment analyses using the “clusterProfiler” R package [22], and the results of adjust *P* value < 0.05 were considered statistically significant.

### 2.4. Construction of a Prognostic FRG Signature

Here, the GSE17679 dataset was randomly divided into train and test cohorts, and the train cohort was used to identify key genes and construct a prognostic FRG signature. We established the gene signature according to the following steps: first, we performed the univariate Cox regression analysis of DEFRGs and selected the genes with *P* value < 0.05 for the subsequent dimension reduction analysis. Next, we used the random survival forest algorithm to further reduce the number of candidate genes. The variable importance (VIMP) and minimal depth are two quantitative indicators calculated by the random survival forest algorithm, which can be used to evaluate the prognostic value of genes. The larger the VIMP or the smaller the minimal depth is, the higher the prognostic value is [23]. In this study, we selected the genes that are not only ranked in the top 15 of VIMP but also ranked in the top 15 of minimal depth for the next dimension reduction analysis. Finally, we performed a multivariate Cox regression analysis, and the genes with *P* value < 0.05 were used to construct the prognostic FRG signature.

### 2.5. Validation of the Prognostic FRG Signature

Patients in the train cohort were given a riskScore. The riskScore was calculated as follows:

(1) $$\begin{array}{c} \text{riskScore}=\overset{n}{\underset{i=0}{\sum }}\beta_{i}*G_{i}, \end{array}$$

where *β*_*i*_ represents the coefficient for gene *i* in the multivariate Cox regression analysis, *G*_*i*_ represents the expression value of gene *i*, and *n* represents the total number of genes in the gene signature.

Based on the median riskScore, patients were divided into high-risk and low-risk groups. Then, the Kaplan-Meier (K-M) survival analysis and the receiver operating characteristic (ROC) curve were utilized to validate the accuracy of the gene signature. Finally, to further evaluate the accuracy and generalization of the gene signature, we also performed the same analysis in the test cohort, the entire GSE17679 cohort, and two independent datasets (GSE63157 and TCGA-SARC cohorts).

### 2.6. Assessing the Potential of the Prognostic FRG Signature in Immunotherapy

A large number of studies indicated that ferroptosis is closely related to tumor immunity [24, 25]. Meanwhile, the results of biological enrichment analysis also showed that DEFRGs are enriched in immune-related pathways. This evidence suggested that the gene signature may have potential application value in immunotherapy for ES, so we conducted an in-depth analysis. First, we studied the difference in immune cell infiltration abundance between distinct risk groups. The abundance of each immune cell infiltration was quantified by the single-sample gene-set enrichment analysis (ssGSEA), and the list of marker gene sets was obtained from a published article [26]. Then, we also did the correlation analysis to study the relationship between 3 key genes and immune infiltrating cells. As a hot topic of immunotherapy, the analysis of immune checkpoints is essential. Therefore, our study also explored the relationship between this gene signature and 5 immune checkpoints (PD-1, PD-L1, CTL4A, LMTK3, and LAG3) [27–29] by performing a correlation analysis and using the STRING (https://string-db.org) which is currently the most comprehensive and authoritative database for exploring protein-protein interactions.

### 2.7. Assessing the Potential of the Prognostic FRG Signature in Chemotherapy

To explore the application value of this gene signature in chemotherapy, we used the “oncoPredict” R package [30], which can predict the half maximum inhibitory concentration (IC_50_) of chemotherapeutic drugs in ES patients according to the data on the Genomics of Drug Sensitivity in Cancer (GDSC) website. Based on this R package, we can not only find the drugs sensitive to ES (IC_50_ < 50) but also explore the sensitivity differences of different drugs in high-risk and low-risk groups, thus contributing to the development of personalized chemotherapy plans for ES patients.

### 2.8. Comprehensive Analysis of Key Genes

To further explore the prognostic value of key genes in ES, we performed a comprehensive analysis. First, we performed K-M survival analysis to explore the effects of high and low expressions of key genes on the prognosis of patients in the GSE17679 cohort. The high and low expression groups were determined by an optimal cut-off point which was calculated by the “survminer” R package. Meanwhile, we also utilized a box plot to observe the differences in the expression levels of the key genes in the normal and tumor tissues. EWSR1 and FLI1 are well-known oncogenes of ES; hence, we performed a correlation analysis between key genes and EWSR1/FLI1 and validated it with the single-cell dataset (GSE130019).

### 2.9. Real-Time Quantitative PCR (RT-qPCR)

After 48 h of cell culture, TRIzol (Invitrogen, USA) was used to isolate total RNA. And the RNA was reverse transcribed into cDNA using a reverse transcriptase kit (TaKaRa, Japan). The expression levels of AURKA, RGS4, and RIPK1 were amplified by real-time fluorescence quantitative PCR (Bio-Rad, USA). GADPH is the internal reference, with 2^-*ΔΔ* CT^ value indicates the relative expression level of target gene mRNA. All primer sequences are as follows:

AURKA: forward primer: 5′-TTCAGGACCTGTTAAGGCTACAGC-3′ and reverse primer: 5′-GAGCCTGGCCACTATTTACAGGT-3′

RGS4: forward primer: 5′-ACATCGGCTAGGTTTCCTGC-3′ and reverse primer: 5′-GTTGTGGGAAGAATTGTGTTCAC-3′

RIPK1: forward primer: 5′-TGGGCGTCATCATAGAGGAAG-3′ and reverse primer: 5′-CGCCTTTTCCATGTAAGTAGCA-3′

GADPH: forward primer: 5′-GGCTGCCCAGAACATCAT-3′ and reverse primer: 5′-CGGACACATTGGGGGTAG-3′

### 2.10. Construction and Evaluation of the Nomogram

At the end of this study, clinical data, including gender, age, state, event, and survival time, were incorporated with the gene signature to construct a nomogram. Meanwhile, calibration curves were also generated to verify the accuracy of the nomogram.

### 2.11. Statistical Analysis

In this study, the student *t*-test and the Wilcoxon signed-rank test were performed to compare differences between groups. The key genes were identified through the univariate Cox regression analysis, the random survival forest algorithm, and the multivariate Cox regression analysis. And these key genes were used to construct the prognostic FRG signature based on the multivariate Cox regression analysis. The K-M curve analysis was used to evaluate the survival differences between patients in different risk groups, and the area under the curve (AUC) value was calculated from the ROC curve using the “timeROC” R package. The R software (version 4.0.3) and GraphPad Prism software (version 8.2.1) were used to perform all data processing. The parameter settings of all R packages used in this study are all default values. And *P* value < 0.05 was considered statistically different unless otherwise specified.

## 3. Results

### 3.1. Identification of Differentially Expressed FRGs and Functional Annotation

**Figure 1:**
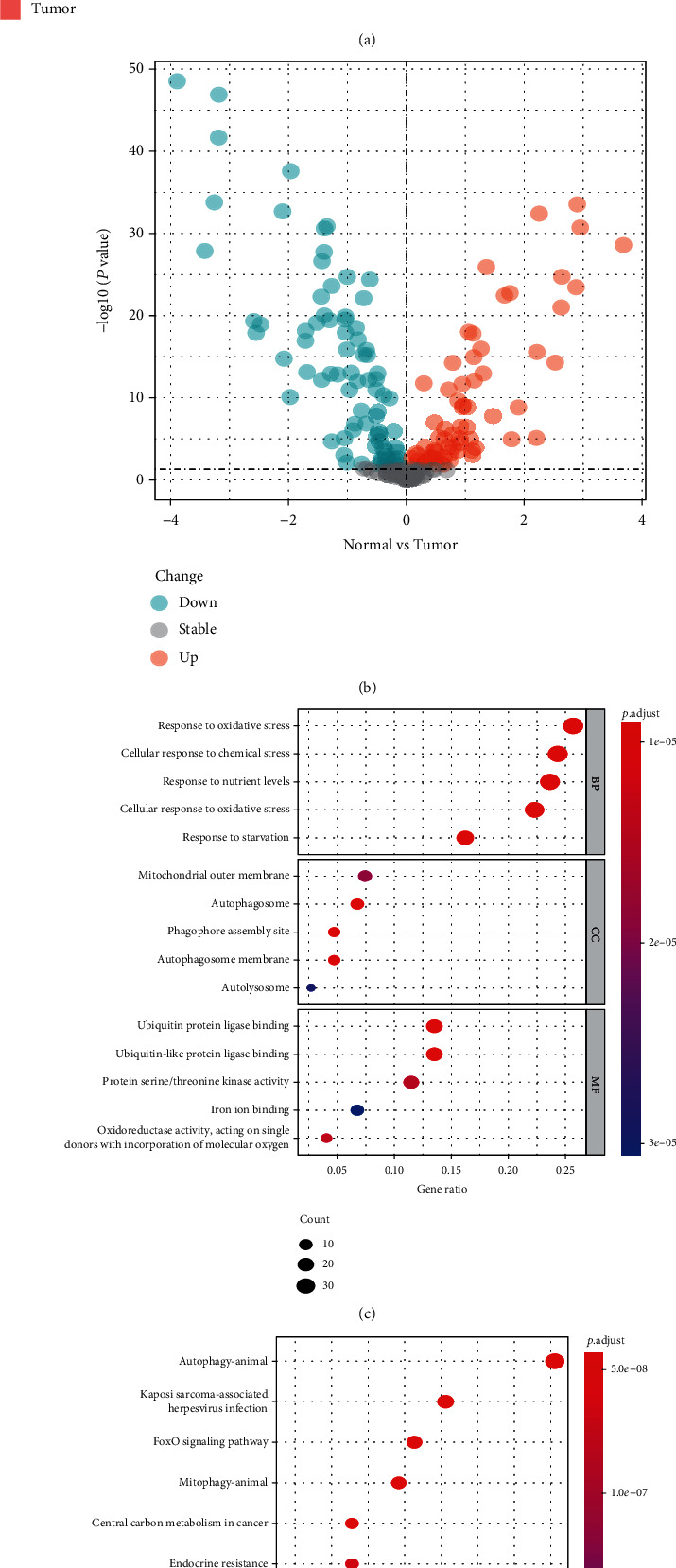
Identification of DEFRGs and functional annotation. (a) A heatmap to show the expression of DEFRGs in normal and tumor tissues. (b) A volcano plot to show the results of differential analysis. (c) GO enrichment analysis for DEFRGs. BP: biological process; CC: cellular component; MF: molecular function. (d) KEGG pathway enrichment analysis for DEFRGs.

As shown in Figures 1(a) and 1(b), 148 genes were identified as DEFRGs, including 67 upregulated genes and 81 downregulated genes. Performing functional annotation analysis of DEFRGs is important for understanding the mechanisms of ferroptosis in ES. Thus, we performed GO and KEGG analyses. The GO analysis results showed that the DEFRGs were mainly involved in response to oxidative stress (BP), mitochondrial outer membrane (CC), and ubiquitin protein ligase binding (MF) (Figure 1(c)). The top five pathways of KEGG analysis results were autophagy-animal, Kaposi sarcoma-associated herpesvirus infection, FoxO signaling pathway, mitophagy-animal, and central carbon metabolism in cancer (Figure 1(d)). All results suggested that the DEFRGs play an important role in tumorigenesis. Furthermore, we also found an intriguing phenomenon that DEFRGs were also enriched in various immune-related signaling pathways, including PD-L1 expression and PD-1 checkpoint pathway in cancer, B cell receptor signaling pathway, T cell receptor signaling pathway, etc. (Table 1). This gave us some insight into establishing a new immunotherapy strategy for ES.

### 3.2. Construction of a Prognostic FRG Signature

**Figure 2:**
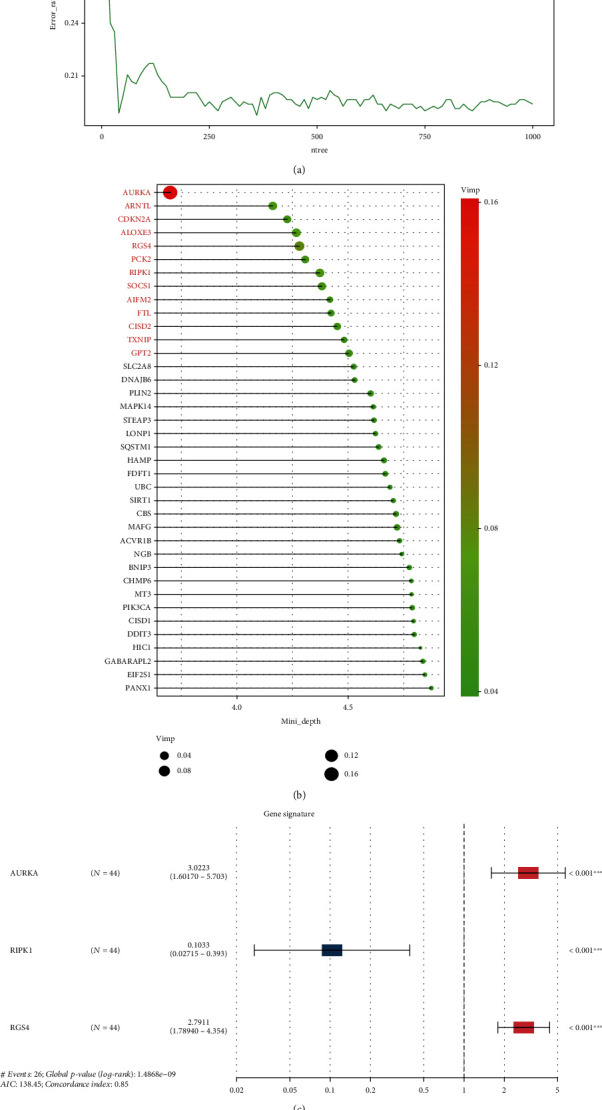
Key FRG selection and gene signature construction process. (a) Estimation of the random forest OOB prediction error rate based on the number of trees. (b) 13 genes ranked in both top 15 VIMP and minimal depth. (c) The forest map composed of 3 independent prognostic characteristic genes.

Here, we selected key prognostic genes from 148 DEFRGs based on univariate Cox regression analysis, random survival forest algorithm, and multivariate Cox regression analysis to construct an accurate prognostic FRG signature. First, we performed preliminary dimensionality reduction for 148 DEFRGs through the univariate Cox regression analysis, and 38 DEFRGs with *P* value < 0.05 (Supplementary Table 1) were selected and used to construct the random survival forest. The out-of-bag (OOB) prediction error plot showed that the random survival forest based on 38 DEFRGs owns a low error rate and excellent stability (Figure 2(a)). This result proved that the 38 DEFRGs indeed had prognostic value. Then, through the random survival forest algorithm, 13 genes were selected for the next dimension reduction analysis. These genes ranked in both the top 15 VIMP and the top 15 minimal depth (Figure 2(b)). Finally, by performing a multivariate Cox regression analysis of the 13 genes (Supplementary Figure 1), we identified 3 key prognostic genes (AURKA, RIPK1, and RGS4) and used them to establish an accurate prognostic FRG signature (Figure 2(c)).

### 3.3. Validation of the Prognostic FRG Signature

**Figure 3:**
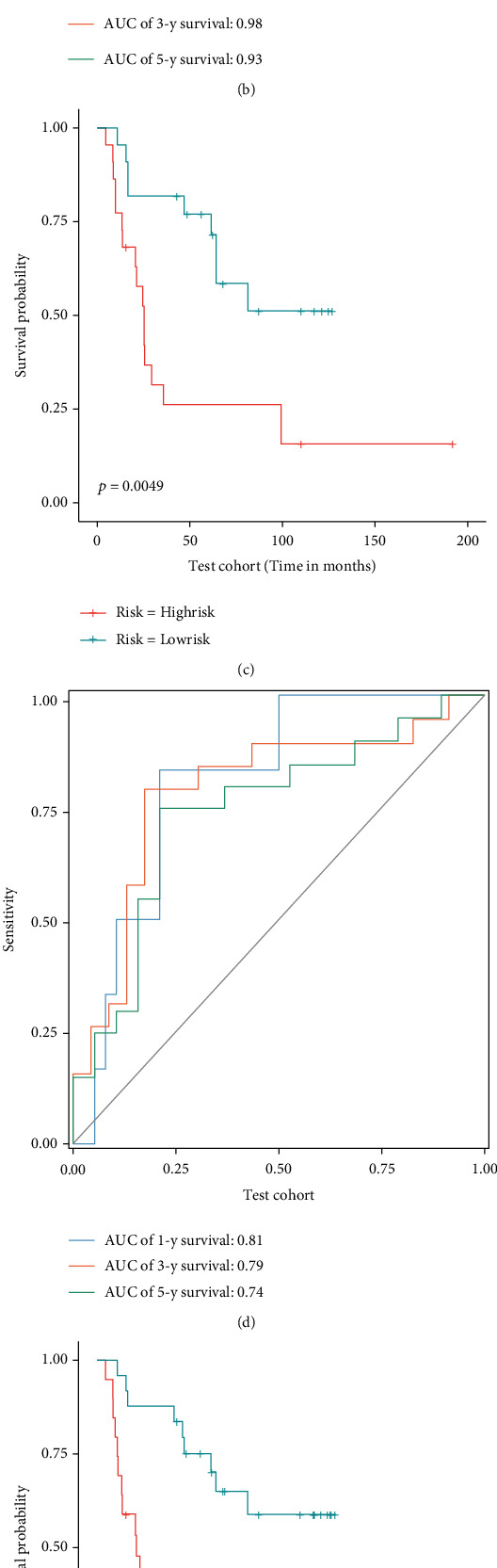
Evaluation of the performance of the gene signature by internal validation cohorts. In train cohort, test cohort, and entire GSE17679 cohort: Kaplan-Meier curves (a, c, e) and ROC curves (b, d, f).

**Figure 4:**
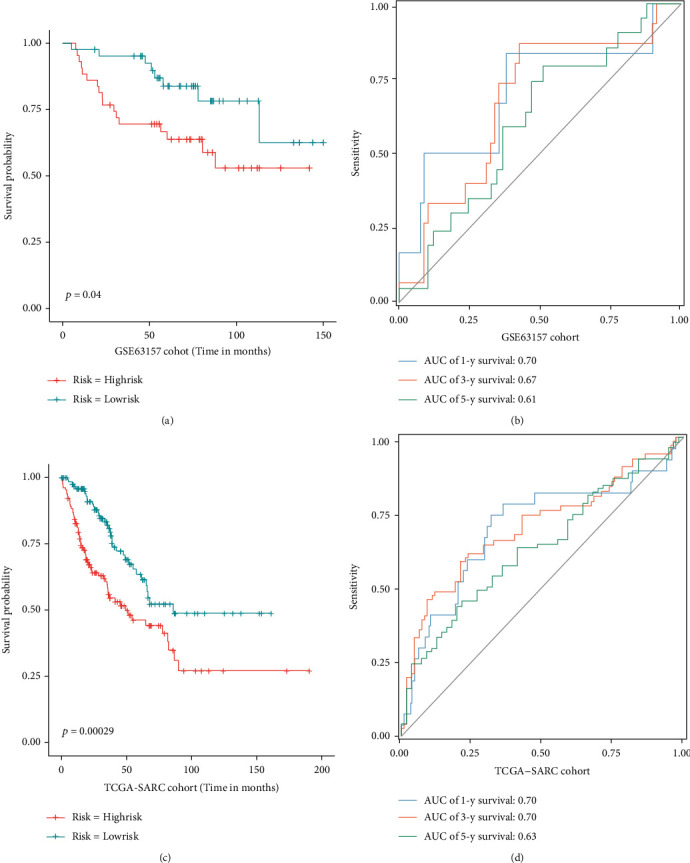
Evaluation of the performance of the gene signature by external validation cohorts. In the GSE63157 cohort and TCGA-SARC cohort: Kaplan-Meier curves (a, c) and ROC curves (b, d).

First, patients were divided into low- and high-risk groups based on median riskScore (the riskScore of each patient = 1.1060 × expression level of AURKA − 2.2701 × expression level of RIPK1 + 1.0264 × expression level of RGS4). Then, we verified the accuracy and generalization ability of the gene signature through the K-M survival analysis and the ROC curve. The validation results of the train cohort showed that patients in the low-risk group lived significantly longer than those in the high-risk group (Figure 3(a)), and the AUC value was 0.90, 0.98, and 0.93 for 1, 3, and 5 years (Figure 3(b)). Meanwhile, the gene signature also showed excellent prediction performance in the test cohort and the entire GSE17679 cohort (Figures 3(c)–3(f)). Furthermore, the validation results by two independent cohorts (GSE63157 and TCGA-SARC) revealed that the generalization ability of the gene signature was also excellent. As shown in Figures 4(a) and 4(c), the results of the K-M survival analysis showed that worse survival was significantly associated with the high-risk group (GSE63157, *P* value = 0.04, and TCGA-SARC, *P* value = 0.00029). And as shown in Figures 4(b) and 4(d), the AUC values of the GSE63157 cohort for predicting 1, 3, and 5 years were 0.70, 0.67, and 0.61, respectively; the AUC values of the TCGA-SARC cohort for predicting 1, 3, and 5 years were 0.70, 0.70, and 0.63, respectively. All results proved that the gene signature has excellent prediction performance and can play a stable predictive prognostic role in different cohorts.

### 3.4. Assessing the Potential of the Prognostic FRG Signature in Immunotherapy

**Figure 5:**
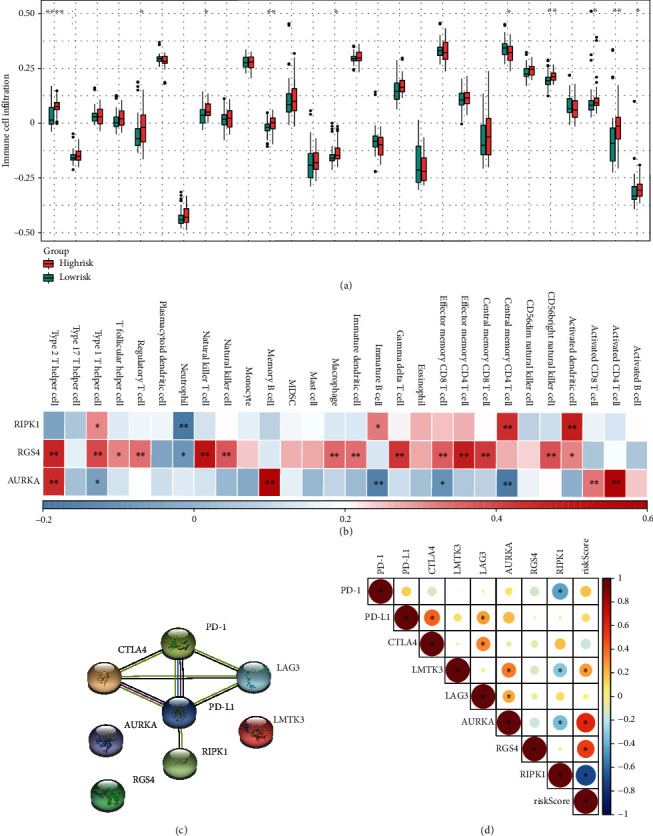
Assessing the potential of the gene signature in immunotherapy. (a) Difference in 28 kinds of immune cell infiltration between high- and low-risk group (^∗^*P* < 0.05; ^∗∗^*P* < 0.01; ^∗∗∗^*P* < 0.001; ^∗∗∗∗^*P* < 0.0001). (b) The correlation between 28 kinds of immune cells and 3 key genes (the *x*-axis label is the same as (a)). (c) PPI network constructed by 5 kinds of immune checkpoint molecules and 3 key genes. (d) Correlation immune checkpoints between riskScore and immune checkpoints/key genes (blue, negative correlation; red, positive correlation; ^∗^*P* < 0.05).

Here, we, respectively, evaluate the landscape of 28 kinds of immune cell infiltration in high- and low-risk samples. As shown in Figure 5(a), we found 10 kinds of immune cells, including activated B cell, activated CD4 T cell, activated CD8 T cell, CD56 bright natural killer cell, central memory CD4 T cell, macrophage, memory B cell, natural killer T cell, regulatory T cell, and type2 T helper cell, presented significant infiltration difference between high- and low-risk groups, and 9 of them were higher infiltration in the high-risk group than those in the low-risk group, except central memory CD4 T cell. And the degree of infiltration of 7 kinds of immune cells has a significant correlation with riskScore (Supplementary Figure 2). The next analysis showed that key genes are closely associated with various immune cells (Figure 5(b)). Furthermore, to explore the relationship between the gene signature and immune checkpoints, we uploaded these genes to the STRING database and performed a correlation analysis. The results showed that RIPK1 and 5 immune checkpoints were in the same protein interaction network (Figure 5(c)). Meanwhile, the results of the correlation analysis also revealed that the 3 key genes and the riskScore all have significant correlations with immune checkpoints (Figure 5(d)). Based on the above results, we have reason to believe that 3 key genes are closely related to tumor immunity, and the gene signature is helpful in improving the immunotherapy for ES.

### 3.5. Assessing the Potential of the Prognostic FRG Signature in Chemotherapy

**Figure 6:**
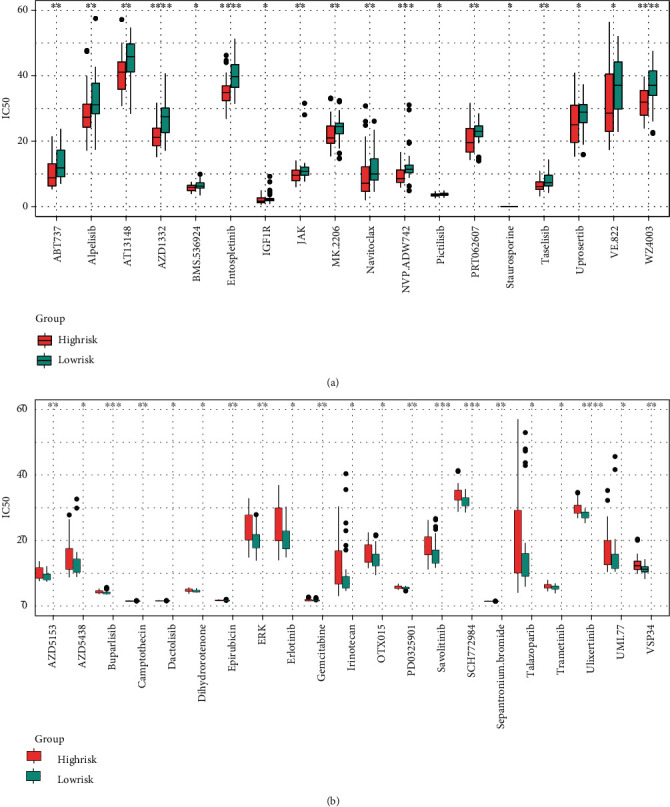
Differences in chemotherapeutic drug sensitivity between high-/low-risk group. (a) Drugs that are more sensitive to high-risk patients. (b) Drugs that are more sensitive to low-risk patients (^∗^*P* < 0.05; ^∗∗^*P* < 0.01; ^∗∗∗^*P* < 0.001; ^∗∗∗∗^*P* < 0.0001).

According to the results of the “oncoPredict” R package, we found that 118 kinds of chemotherapeutic drugs were sensitive to ES (Supplementary Figure 3), and 39 of them showed significant differences between the high- and low-risk groups. As shown in Figures 6(a) and 6(b), patients in the high-risk group were more sensitive to 18 types of chemotherapeutic drugs than those in the low-risk group, and patients in the low-risk group were more sensitive to 21 types of drugs. This result demonstrated that the gene signature was also helpful for the formulation of a personalized chemotherapy strategy for ES.

### 3.6. Comprehensive Analysis of Key Genes

**Figure 7:**
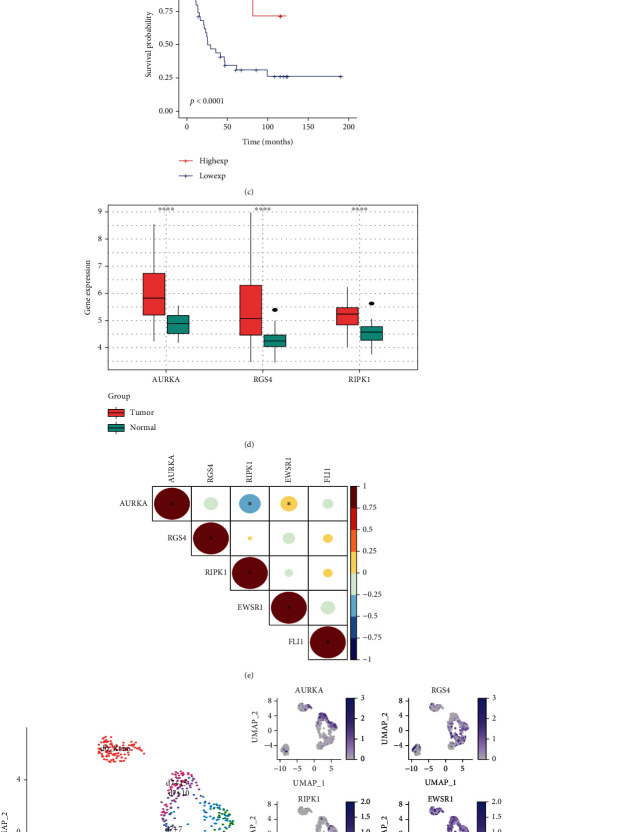
Comprehensive analysis of key genes. (a–c) Survival analyses of the 3 key genes. (d) Different expression of 3 key genes between tumor and normal tissue (^∗^*P* < 0.05; ^∗∗^*P* < 0.01; ^∗∗∗^*P* < 0.001; ^∗∗∗∗^*P* < 0.0001). (e) Correlation analysis between 3 key genes and EWSR1/FLI1 (^∗^*P* < 0.05). (f) Single-cell analysis: effect of dynamic changes in ERWR1/FLI1 expression on the expression of 3 key genes (Xneo: A673 cell xenografts in a severe combined immunodeficiency mouse. In a UAMP plot: one point represents a cell; the darker the color of the point, the higher the gene expression. The points on all UAMP plot are positioned consistently, and the UAMP plot on the left is used as the reference diagram).

As shown in Figures 7(a)–7(c), we found that in the GSE17679 cohort, the low expression group of AURKA or RGS4 survived longer than their high expression group; the survival time of RIPK1 high expression group was longer than the low expression. We also found that all 3 key genes were significantly highly expressed in tumor tissue (Figure 7(d)). Additionally, correlation analysis revealed that there was a significant positive correlation between AURKA and EWSR1 (Figure 7(e)). The subsequent single-cell analysis also confirmed this result. As shown in Figure 7(f), during the continuous period of continuous using doxycycline for 7 days (d0_Xeno-d7_Xeno), the expression of EWSR1 was inhibited and gradually decreased, while the expression of AURKA also showed a decreasing trend. Subsequently, during the period of 15 days after discontinuation of the drug (d7-d7+15), the expression of EWSR1 started to recover, and the expression of AURKA also increased with increasing expression of EWSR1. Thus, a close relationship does exist between EWSR1 and AURKA. In conclusion, our study showed that all 3 key genes play key roles in tumor immunity and prognosis of ES, among which AURKA is also closely related to the oncogenic process of ES. Therefore, they are likely to be potential therapeutic targets for ES.

### 3.7. Validation of Expression Level of Three Key Genes in ES Cell

**Figure 8:**
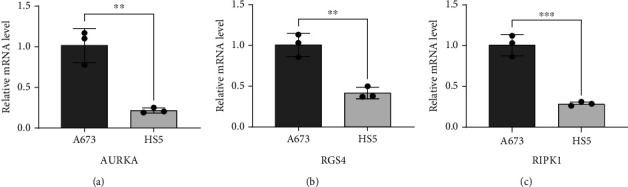
Real-time quantitative PCR (RT-qPCR). Validation of differential expression of 3 key FRG mRNAs between tumor and normal cells. (a) AURKA; (b) RGS4; (c) RIPK1 (^∗∗^*P* < 0.01; ^∗∗∗^*P* < 0.001).

The results of the above analysis showed that 3 key genes were highly expressed in ES. In order to verify that the 3 genes are specifically highly expressed in ES tissue, we performed RT-qPCR in the ES (A673) and normal bone marrow stroma (HS5) cell lines. As shown in Figures 8(a)–8(c), the expression of AURK1, RGS4, and RIPK1 in A673 was significantly higher than in HS5. Hence, this cell experiment further verifies the reliability of the results of this bioinformatics analysis.

### 3.8. Construction and Evaluation of the Nomogram

**Figure 9:**
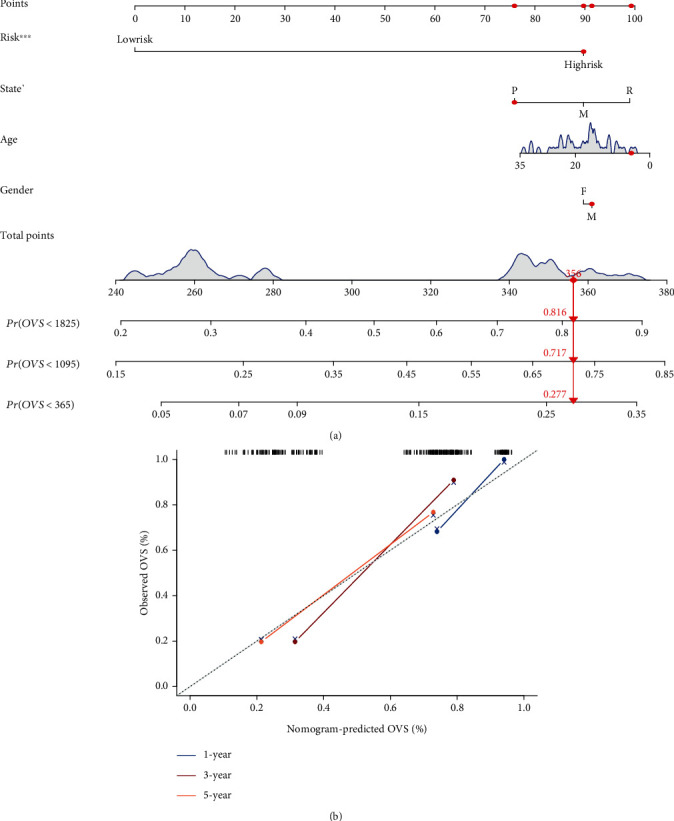
Nomogram plot and calibration curves. (a) Nomogram for predicting the 1-, 3-, and 5-year survival probability of patients with ES (state: P: primary; R: recurrence; M: metastasis). (b) Calibration curves for the nomogram at 1, 3, and 5 years (OVS: overall vital survival). The *Y* axis represents the actual OVS while the *X* axis represents the predicted OVS.

Based on the clinical data and the gene signature, we constructed a comprehensive prognostic nomogram that can help clinicians estimate the probability of survival for 1, 3, and 5 years in ES patients (Figure 9(a)). Meanwhile, the calibration curves of 1-, 3-, and 5-year survival showed that the predicted results of the nomogram were highly consistent with the actual results (Figure 9(b)), which demonstrated that the nomogram was accurate.

## 4. Discussion

Currently, ES patients with localized disease have a 5-year survival rate of 70–80%, but those with metastases have a significantly lower survival rate of <30%. Regrettably, more often than not, approximately 20–25% of ES patients present with metastases at diagnosis [4]. Patients who cannot be surgically treated have no choice but to receive systemic treatments. However, side effects and treatment failures of common systemic treatments (including chemotherapy and targeted therapies, etc.) are also frequently reported [31, 32]. Combined with no robust statistical tool to estimate the prognosis of ES patients, the therapy of ES remains a challenge. It urges us to develop novel therapeutic strategies. Ferroptosis, which is a form of programmed cell death independent of apoptosis, is modulated by several pathways and is closely related to various diseases [33]. Recent evidence has indicated that ferroptosis is closely related to tumorigenesis, the tumor suppression process, and the treatment response [34]. Meanwhile, we also found that many FRGs are abnormally expressed in ES tissues, and most of them were related to prognosis. Therefore, we carried out this research to find potential biomarkers from FRGs and develop a prognostic FRG signature to improve the prognosis of patients with ES.

In this study, we first did the differential expression analysis of genes between normal and ES tissues and screened out 148 DEFRGs. Then, we did the GO and the KEGG analyses of DEFRGs, and the results indicated that 148 DEFRGs play an important role in tumorigenesis. And we also found that these genes were also enriched in immune-related pathways, which provide a new approach to immunotherapy for ES. Hence, DEFRGs deserve further analysis.

We used three analysis methods step by step (univariate Cox regression analysis, random survival forest algorithm, and multivariate Cox regression analysis) to identify key genes and used the identified genes to construct the gene signature. In the construction process of the gene signature, we did not adopt LASSO regression analysis but adopted the random survival forest algorithm for the following reasons. First, two forms of randomization were combined, including case resampling and variable subsetting, which made the prediction results robust and accurate [23]. Second, two quantitative indicators, VIMP, and minimal depth could help with key gene selection. The random survival forest algorithm also has some disadvantages. It did not provide explicitly formatted formulas and was unable to extract information regarding the underlying process. Using genes screened by random survival forest directly to construct the gene signature is not reliable, so that is the reason why we did further multivariate regression analysis. As expected, the gene signature composed of 3 genes showed favorable performance in both internal and external validation cohorts. Next, we performed an in-depth analysis to further explore the application potential of this gene signature.

Tumor immunoediting theory proposed that less immunogenic cancer cells are selected during tumor development to evade antitumor immune responses [35, 36]. Hence, the tumors have several immunosuppressive mechanisms, such as increased immunosuppressive cells (regulatory T cells and tumor-associated macrophages), decreased expression of cancer antigens, and increased expression of immune checkpoints (CTL4A and PD-1) [37, 38]. In our study, we found that high-risk ES patients generally had higher regulatory T cell and macrophage infiltration than low-risk patients. As the most classical immune checkpoint, PD-1 is a central regulator of CD8+ T cell exhaustion, whose overexpression can inhibit T cell immunity in several different types of cancers [39, 40]. Our study found that RIPK1 and PD-1 were not only in the same PPI network but also significantly negatively correlated. Furthermore, the 3 key genes were also closely related to other different immune checkpoints. Combined with the results of comprehensive analysis and cell experiments of the 3 genes, we believe that this gene signature and the 3 key genes have a bright future in ES therapy. Hence, the 3 genes deserve further study.

Aurora kinase A (AURKA) belongs to the family of serine/threonine kinases, which play essential roles in regulating cell division during mitosis and has been a popular target. Numerous studies have reported that AURKA was function as oncogenes to promote tumorigenesis in multiple types of cancer, including lung cancer [41], gastric cancer [42], and pancreatic cancer [43]. Although AURKA has attracted the attention of many researchers, few studies have focused on the mechanism of AURKA in ES. To our knowledge, ES is characterized by a recurrent balanced chromosomal translocation [44], which results in the fusion of genes from the FET family and genes from the ETS family. One study has reported that transcription of AURKA is positively regulated by E4TF1, which is a ubiquitously expressed ETS family protein [45]. And in our study, we also found a significant correlation between AURKA and EWSR1. This reveals that AURKA is closely related to the carcinogenic process of ES. Furthermore, a review study has indicated that AURKA can become a target for cancer therapy and found that most tumor types show significantly higher AURKA expression than normal tissue in the TCGA database. Meanwhile, AURKA inhibitors (AKIs) have been tested in preclinical studies, and some of them have been subjected to clinical trials as monotherapies or in combination with classic chemotherapy or other targeted therapies [46]. Therefore, there is sufficient evidence that AURKA plays an important role in the tumorigenesis of ES and can be a promising therapeutic target. Hence, it deserves further study.

Regulators of G-protein signaling (RGS) proteins are G-protein-coupled receptors (GPCRs) mediated response regulators in cells, which are important drug targets for malignant tumors [47, 48]. To date, more than 20 proteins from the RGS family have been identified. One of them is RGS4, a negative regulator of GPCR that can block the transmission of related signaling factors by accelerating G-protein proteolysis. Many studies have reported that RGS4 is associated with increased cancer cell viability, invasion, and/or motility in glioma [49], triple-negative breast cancer [47], etc. However, the role of RGS4 remains poorly understood in ES, and the related mechanism remains to be further explored.

Regarding receptor-interacting kinase 1 (RIPK1), it is a key node in the TNF signal transduction pathway, actively controlling the balance between gene activation and induction of cell death in the form of apoptosis and necroptosis [50]. RIPK1 has now emerged as an important drug target not only due to its key roles in TNF signaling but also because its kinase structure is highly amenable to the development of specific small molecule pharmacological inhibitors [51]. Many studies have reported that RIPK1 inhibitors present an opportunity to treat a range of human degenerative and inflammatory diseases, including colitis, dermatitis, traumatic brain injury, stroke, lysosomal storage diseases, amyotrophic lateral sclerosis (ALS), and multiple sclerosis (MS) [52, 53]. However, studies associated with cancer show that RIPK1 is a gene with “two faces” in both pro- and anticarcinogenic functions [54]. In head and neck squamous cell carcinoma and liver cancer, the low expression of RIPK1 is associated with poor prognosis, while in breast cancer (BC) and glioblastoma, the high expression of RIPK1 leads to poor prognosis. In this study, we found that patients with high RIPK1 expression lived longer than those with low expression, and the expression of RIPK1 was positively correlated with a variety of antitumor immune cells (activated dendritic cell and type 1 T helper cell) and was negatively correlated with a variety of immune checkpoints (especially PD-1). Therefore, for ES, RIPK1 is an important anticarcinogenic gene, and its high expression can enhance anticancer immunity and prolong the survival time of ES patients. An in-depth study of this gene may lead to new options for ES treatment.

Chemotherapy has been the most commonly used cancer treatment, but failures and side effects of treatment are frequently reported [55, 56]. Thereby, the selection of sensitive chemotherapeutic drugs is critical for cancer treatment. In our study, we not only found 118 agents which were sensitive to ES but also found that 39 agents displayed significantly sensitive differences between high- and low-risk patients. Among them, 18 kinds of chemotherapeutic drugs were more sensitive to patients in the high-risk group, while the other 21 drugs were more sensitive to those in the low-risk group. Thus, this gene signature is also helpful for the formulation of a personalized chemotherapy strategy for ES patients.

At the end of this study, we created a visual nomogram quantitatively assessing the overall survival of ES patients based on the gene signature and clinical characteristics. And the results of the calibration curve suggest that the nomogram was reliable. Therefore, our study also provides a reliable and practical statistical tool which can help clinicians assess the survival time of patients with ES.

Of course, there are several limitations to our study. First, since ferroptosis has always been a hot area, subsequent studies may report more FRGs than just the 205 genes in this study. Second, due to the limited clinical data provided by public datasets, the study was unable to incorporate a sufficient number of clinical characteristics, which may result in potential biases in the performance of the gene signature. Third, due to different sequencing platforms, the expression levels of all genes are relative values, which brings difficulties to the determination of absolute thresholds in clinical practice.

## 5. Conclusions

In conclusion, by exploring the mechanism of ferroptosis in ES, we identified 3 key genes significantly associated with the tumor immunity and prognosis of ES patients and used them to establish a prognostic FRG signature. Subsequent analysis showed that 3 key genes are potential therapeutic targets, and the established gene signature could act as a robust and independent biomarker for predicting patient prognosis and contribute to the development of more effective immunotherapy and chemotherapy strategies. In addition, we also constructed an accurate nomogram, which contributes to clinicians evaluating the survival time of ES patients. Therefore, our study helps improve the prognosis of patients with ES.

## Figures and Tables

**Table 1:** Immune-related signaling pathways in the results of KEGG analysis.

ID	Description	Gene	Log10 (adj. *P*)
hsa05235	PD-L1 expression and PD-1 checkpoint pathway in cancer	HIF1A, MAPK1, EGFR, KRAS, HRAS, NRAS, MAPK14, PIK3CA	-3.733
hsa04662	B cell receptor signaling pathway	MAPK1, KRAS, HRAS, NRAS, PIK3CA	-1.865
hsa04660	T cell receptor signaling pathway	MAPK1, MAPK8, KRAS, HRAS, NRAS, MAPK9, MAPK14, PIK3CA	-3.346
hsa05166	Human T-cell leukemia virus 1 infection	TP53, MAPK1, SLC2A1, MAPK8, KRAS, CDKN2A, HRAS, NRAS, TGFBR1, MAPK9, ZFP36, CDKN1A, PIK3CA	-4.08
hsa04657	IL-17 signaling pathway	MAPK1, MAPK8, ELAVL1, TNFAIP3, MAPK9, MAPK14	-2.249
hsa04659	Th17 cell differentiation	HIF1A, MAPK1, MAPK8, TGFBR1, MAPK9, MAPK14	-1.997
hsa04658	Th1 and Th2 cell differentiation	MAPK1, MAPK8, MAPK9, MAPK14	-1.127

## Data Availability

The data were obtained from GEO (https://www.ncbi.nlm.nih.gov/geo/) and UCSC Xena (https://xena.ucsc.edu/).

## Supplementary Materials

Supplementary 1 Supplementary Figure 1: results of multivariate Cox regression analysis for 13 genes.

Supplementary 2 Supplementary Figure 2: the correlation between riskScore and 7 kinds of immune cell infiltration (blue, negative correlation; red, positive correlation).

Supplementary 3 Supplementary Figure 3: 118 chemotherapy drugs sensitive to ES.

Supplementary 4 Supplementary Table 1: results of univariate Cox regression analysis with *P* < 0.05.
