# Supplementary figures and images for: A Novel Ferroptosis-Related Gene Signature for Prognosis Prediction in Ewing Sarcoma
Source: Anal Cell Pathol (Amst). 2022 Aug 22;2022:6711629. doi: 10.1155/2022/6711629 (PMC9425108; doi:10.1155/2022/6711629)

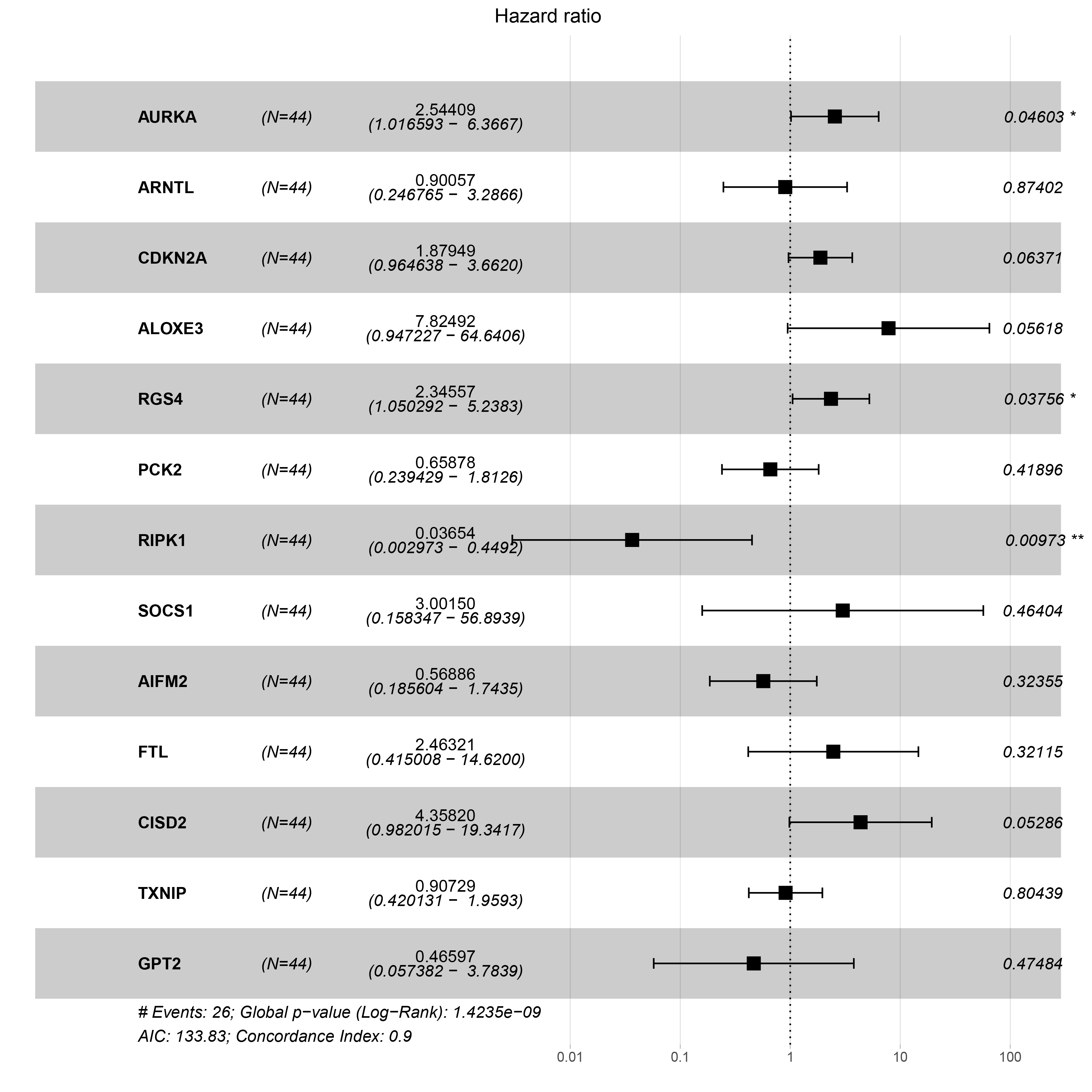

Supplement: Supplementary 1 — Supplementary Figure 1: results of multivariate Cox regression analysis for 13 genes. [file 6711629.f1.png]

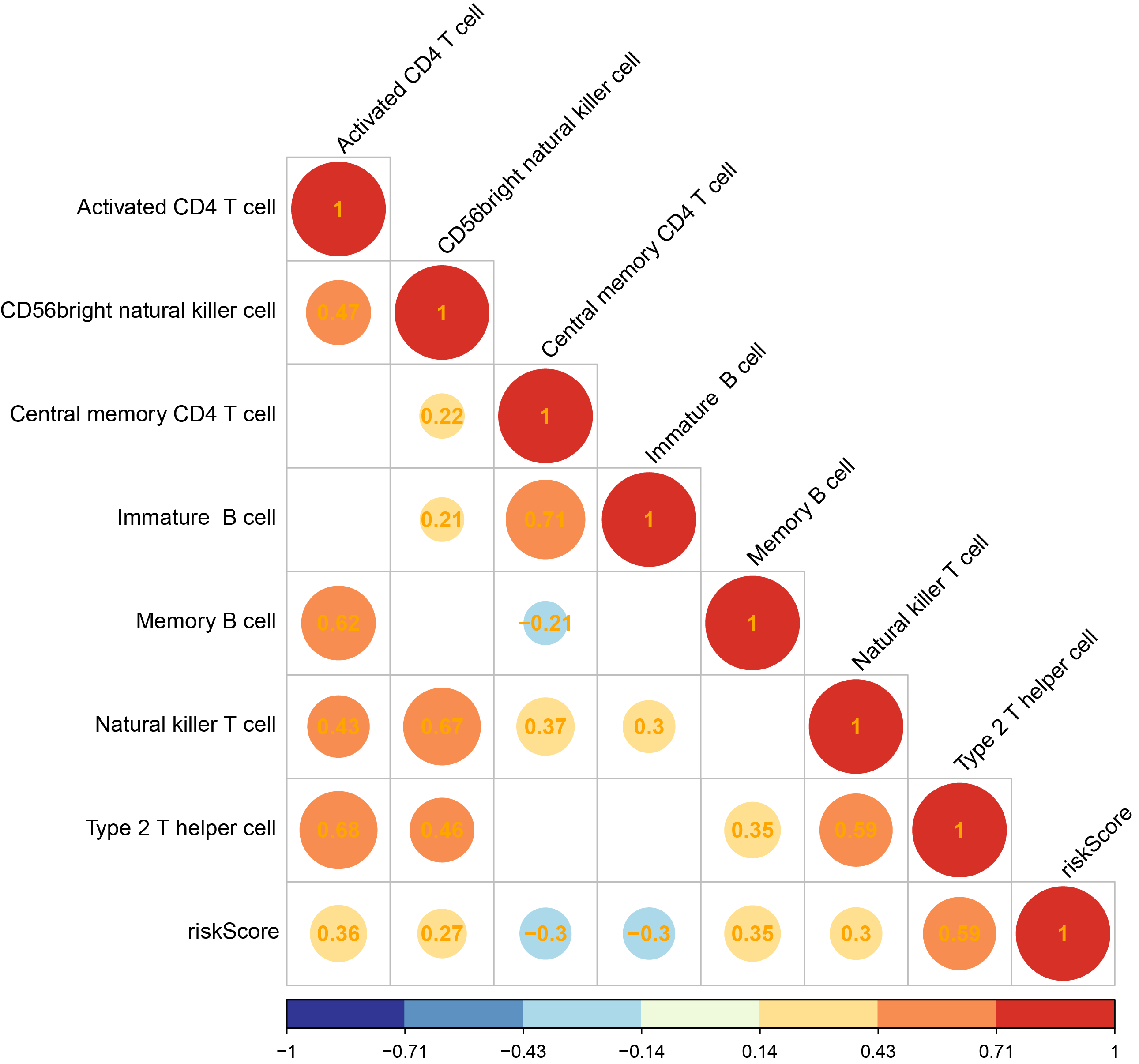

Supplement: Supplementary 2 — Supplementary Figure 2: the correlation between riskScore and 7 kinds of immune cell infiltration (blue, negative correlation; red, positive correlation). [file 6711629.f2.png]
