# Supplementary material for: A Novel Ferroptosis-Related Gene Signature for Prognosis Prediction in Ewing Sarcoma
Source: Anal Cell Pathol (Amst). 2022 Aug 22;2022:6711629. doi: 10.1155/2022/6711629 (PMC9425108; doi:10.1155/2022/6711629)
Supplement: Supplementary 4 — Supplementary Table 1: results of univariate Cox regression analysis with P < 0.05. [file 6711629.f4.docx]

| **Gene** | **HR** | **95% CI** | **-Log10(P.value)** |
| --- | --- | --- | --- |
| **GPT2** | 3.46 | 1.29-9.29 | 1.86 |
| **CISD1** | 1.86 | 1.04-3.32 | 1.43 |
| **MAFG** | 3.99 | 1.64-9.72 | 2.63 |
| **DNAJB6** | 2.12 | 1.19-3.78 | 1.96 |
| **CHMP6** | 6.74 | 1.78-25.56 | 2.3 |
| **SLC2A8** | 9.64 | 2.11-44.07 | 2.46 |
| **FTL** | 2.14 | 1.06-4.32 | 1.47 |
| **SQSTM1** | 0.48 | 0.27-0.84 | 1.99 |
| **UBC** | 0.19 | 0.04-0.9 | 1.43 |
| **GABARAPL2** | 0.32 | 0.14-0.74 | 2.12 |
| **MT3** | 5.24 | 1.47-18.71 | 1.97 |
| **PCK2** | 0.47 | 0.24-0.94 | 1.48 |
| **HAMP** | 11.19 | 2.53-49.47 | 2.84 |
| **TXNIP** | 0.6 | 0.39-0.9 | 1.84 |
| **CISD2** | 3.57 | 1.31-9.74 | 1.89 |
| **ALOXE3** | 8.46 | 2.68-26.78 | 3.55 |
| **CDKN2A** | 2.08 | 1.34-3.22 | 2.97 |
| **AIFM2** | 0.42 | 0.23-0.77 | 2.31 |
| **RIPK1** | 0.18 | 0.07-0.42 | 4.05 |
| **ARNTL** | 0.39 | 0.19-0.82 | 1.91 |
| **EIF2S1** | 0.31 | 0.11-0.85 | 1.65 |
| **SIRT1** | 0.35 | 0.15-0.8 | 1.88 |
| **PANX1** | 2.59 | 1.01-6.66 | 1.31 |
| **CBS** | 1.74 | 1.19-2.55 | 2.35 |
| **AURKA** | 2.26 | 1.51-3.37 | 4.15 |
| **BNIP3** | 0.65 | 0.48-0.89 | 2.18 |
| **ACVR1B** | 1.98 | 1.03-3.8 | 1.39 |
| **RGS4** | 1.38 | 1.01-1.89 | 1.35 |
| **STEAP3** | 0.51 | 0.31-0.84 | 2.08 |
| **PLIN2** | 1.59 | 1.17-2.16 | 2.51 |
| **HIC1** | 8.41 | 1.42-49.79 | 1.72 |
| **LONP1** | 3.41 | 1.55-7.49 | 2.63 |
| **NGB** | 7.79 | 1.74-34.8 | 2.14 |
| **SOCS1** | 0.07 | 0.01-0.48 | 2.15 |
| **MAPK14** | 0.34 | 0.13-0.88 | 1.59 |
| **FDFT1** | 1.96 | 1.01-3.82 | 1.33 |
| **PIK3CA** | 0.43 | 0.26-0.73 | 2.74 |
| **DDIT3** | 2.09 | 1.13-3.87 | 1.73 |

**Supplementary Table 1：Results of univariate COX analysis with P < 0.05.**
